# Adaptive Temperature Control of Air Conditioners Based on Millimeter-Wave Radar and Light Gradient Boosting Machine Model

**DOI:** 10.3390/s26175428

**Published:** 2026-08-27

**Authors:** Yunlong Xia, Zuoting Song, Wanna Zhang, Zhe Shang, Zhuoqi Zeng, Amr Alanwar

**Affiliations:** 1 Department of Computer Engineering, TUM School of Computation, Information and Technology, Technical University of Munich, Heilbronn Campus, 74076 Heilbronn, Germany; yunlong.xia@tum.de; 2 Midea Group, Shanghai 201702, China; 3 Shanghai Nearlink Open Lab, Shanghai 201702, China; 4 School of Engineering, Hainan Bielefeld University of Applied Sciences, Haikou 570100, China

**Keywords:** millimeter-wave radar, bed localization, air conditioning, adaptive temperature control, LGBM, temperature fluctuation suppression, thermal comfort

## Abstract

**Highlights:**

**What are the main findings?**

- A millimeter-wave radar perception scheme is adopted to accurately capture indoor human presence and activity information, solving the problem of inaccurate air conditioner temperature control caused by traditional single-sensor monitoring.
- An adaptive air conditioner temperature control model based on the Light Gradient Boosting Machine (LGBM) is constructed to realize intelligent prediction and dynamic adjustment of the optimal indoor temperature.

**What are the implications of the main findings?**

- The proposed method effectively improves indoor thermal comfort and reduces invalid energy consumption of air conditioning systems, balancing living comfort and energy efficiency.
- The intelligent control strategy integrating radar perception and machine learning provides a feasible technical reference for the intelligent and energy-saving transformation of building air conditioning systems.

**Abstract:**

To address the issues of large temperature fluctuations, poor spatial perception, and low control robustness in traditional residential air conditioners, this paper proposes an adaptive temperature control algorithm based on millimeter-wave radar and a Light Gradient Boosting Machine (LGBM). Given that the bed is the primary obstacle and heat source in a bedroom, we develop a bed localization method using point cloud clustering. This method accurately identifies the bed position through time-window filtering, outlier removal, and density clustering. An LGBM weak teacher model, trained on massive cloud data, takes the bed position, indoor temperature, and compressor parameters as inputs to optimize air direction and fan speed, thereby effectively suppressing steady-state fluctuations in the return air temperature. Experiments on 719 real-world devices demonstrate that the bed positioning localization consistency rate reaches 83.6% under an error tolerance of 0.5 m, the average absolute temperature fluctuation is reduced to 0.21 °C, and the control accuracy of the air guide mechanism exceeds 0.98. The proposed method requires no hardware modification, offers strong generalizability and low deployment cost, significantly improves temperature stability and thermal comfort in bedroom environments, and provides a feasible technical solution for intelligent residential air conditioning control.

## 1. Introduction

**Figure 1:**
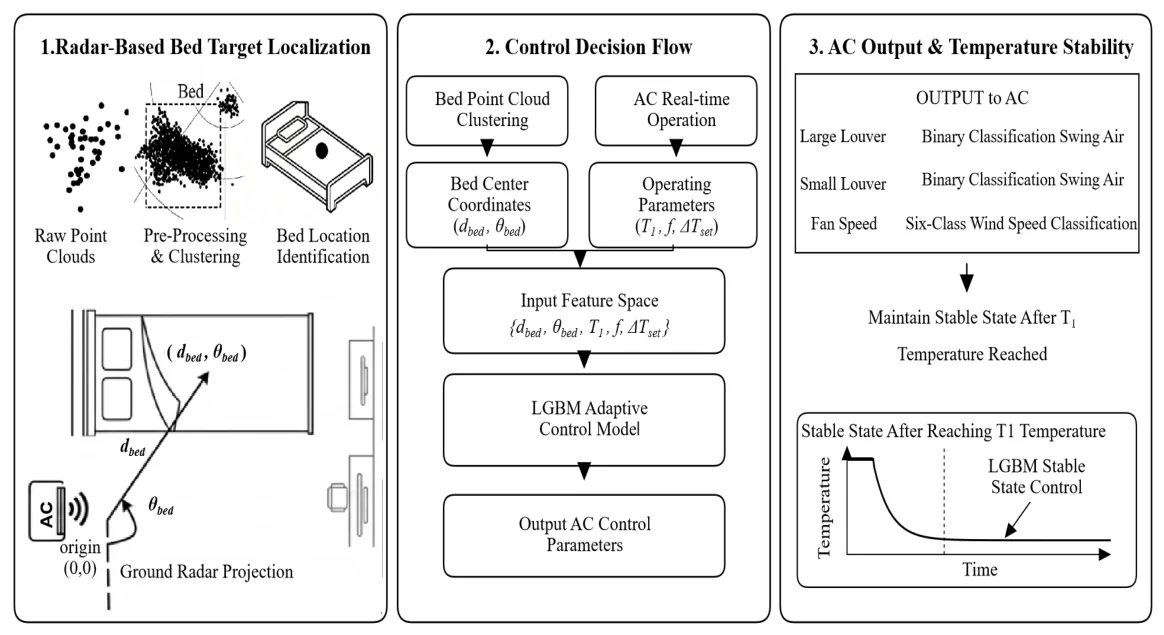
Overall workflow of the millimeter-wave (mmWave) radar-based system. The system is primarily divided into two core components: bed localization and air conditioner control. The system utilizes radar point cloud clustering to obtain the bed positioning parameter pair $\left(d_{bed},\theta_{bed}\right)$. Here, $d_{bed}$ is defined as the horizontal radial distance between the ground projection point of radar and the center of the bed, and $\theta_{bed}$ stands for the horizontal azimuth angle of the bed center with respect to the radar ground projection point. Combined with $T_{1}$, *f* and $\Delta T_{\mathrm{set}}$ acquired from the air conditioner terminal, the above localization outputs form a total of five parameters, which serve as the direct input features of the Light Gradient Boosting Machine (LGBM) model. For detailed information on the model input parameters, refer to *Input Feature Space* in Section 3.2.3. This establishes a closed-loop regulation framework that dynamically adjusts the louver angle and fan speed to suppress indoor temperature fluctuations.

Air conditioning is an indispensable component in modern buildings, which enables control over indoor air temperature, humidity, airflow velocity, and direction to provide a satisfactory thermal environment for occupants [1]. As one of the most energy-intensive electrical systems in buildings, the operating mode of air conditioners directly affects building energy efficiency, as well as indoor thermal comfort and work efficiency [2,3] (Figure 1).

According to compressor control strategies, air conditioners are classified into fixed-frequency and variable-frequency types. Fixed-frequency air conditioners adopt fixed-speed compressors and regulate temperature by switching the compressor on and off [4]. This operation leads to large indoor temperature fluctuations, compromising both comfort and energy efficiency. Due to operational limitations, fixed-frequency air conditioners are mostly applied in small spaces with limited budgets, short-term usage, or stable thermal loads [5,6]. Variable-frequency air conditioners use inverters to convert grid-frequency alternating current into adjustable-frequency power supplies, enabling dynamic matching of cooling or heating capacity according to real-time indoor loads [7]. This reduces temperature fluctuation ranges and extends fluctuation periods, significantly improving temperature control precision and thermal comfort. Studies show that variable-frequency air conditioners maintain stable control accuracy under high ambient temperatures, whereas temperature fluctuations of fixed-frequency units can increase to ±2.0–3.0 °C, enlarging the comfort gap between the two types [8]. With advantages of energy saving, low noise, and precise temperature control, variable-frequency air conditioners are widely used in scenarios requiring long-term continuous operation, high thermal comfort, or large load variations [9].

Both types of air conditioners exhibit temperature fluctuations after reaching the setpoint, with differing ranges depending on operation modes [10]. Fixed-frequency air conditioners typically cause fluctuations exceeding 2 °C due to their working principle, while variable-frequency units limit fluctuations to within 1 °C. Such fluctuations degrade both the energy efficiency of air conditioning systems and occupant thermal comfort [11]. Reducing steady-state temperature fluctuations after setpoint attainment has become an important research topic in air conditioning control. Existing solutions fall into three categories:

- Hardware upgrading: Optimizing core components such as compressors and throttling parts. Variable-frequency air conditioners equipped with DC inverters and electronic expansion valves can restrict steady-state fluctuations to ±0.8–0.9 °C [12]; precision air conditioners using modulated compressors and multi-sensor arrays achieve accuracy of ±0.1–0.3 °C [13]. However, hardware modification is impractical for residential applications.
- Operation mode optimization: Strategies including “rapid cooling and steady-state fine-tuning,” upward air supply, and auxiliary circulation fans to balance indoor airflow without hardware changes [14,15]. These methods rely on manual operation and improve perceived comfort but do not fundamentally eliminate temperature fluctuations.
- Control algorithm optimization: Intelligent algorithms, such as fuzzy neural network-based PID, enhance adaptive control and mitigate disturbances from occupancy and solar radiation [16]; centralized air conditioners combined with load sensing and group control stabilize temperature and reduce energy consumption [17]. However, these algorithms require training data, have poor cross-building generalization, and depend on high-precision sensors and dedicated controllers, leading to high deployment costs [18,19].

To tackle the drawbacks of high hardware expenditure, reliance on manual operation and poor generalization of conventional algorithms, this paper presents an adaptive temperature control algorithm for air conditioners based on millimeter-wave radar sensing, as shown in Section 1. Radar point cloud data are utilized to identify the bed area, and the air supply speed and direction are dynamically tuned according to bed coordinates to mitigate steady-state indoor temperature fluctuations.

Compared with existing strategies, the proposed method possesses three advantages: (1) favorable generalization without scene-specific model retraining; (2) no requirement for core air conditioner hardware reconstruction, leading to low deployment barriers and controllable costs; (3) fully automatic non-intrusive regulation that reduces user manipulation and enhances indoor thermal comfort.

The remainder of this paper is organized as follows. Section 2 defines the core technical challenges. Section 3 describes the bed localization algorithm and LGBM control model. Section 4 presents experimental results. Section 5 analyzes limitations of this work, and Section 6 draws the conclusions.

## 2. Problem Statement

Although variable-frequency air conditioners substantially mitigate temperature fluctuations compared with fixed-frequency units, steady-state oscillations of return air temperature $T_{1}$ persist in bedroom scenarios after reaching the setpoint. Existing solutions, including hardware upgrading, manual adjustment, and intelligent control strategies, suffer from high costs, user dependence, or poor cross-room generalization [12,13,14,15,16,17]. Most importantly, none of these methods address the essential cause of temperature oscillation: bed-induced spatial airflow inhomogeneity.

In bedroom environments, the bed acts as both the dominant airflow obstacle and continuous human heat source [20]. It distorts the propagation and velocity distribution of supplied air, forming recirculation and dead zones that block effective air circulation toward the return vent. During rest, the human body produces a stable metabolic heat flux of 40–50 ${W/m}^{2}$, establishing a persistent thermal boundary layer around the bed [21]. Occasional body movements further disturb thermal distribution and introduce unpredictable transient thermal loads [21]. Such dual effects induce continuous $T_{1}$ fluctuations even under stable compressor operation, since the sensor-measured temperature near the indoor unit cannot fully reflect the actual thermal condition of the occupied zone.

Conventional air conditioners rely purely on built-in temperature feedback without spatial perception of bed position. Without awareness of the relative positional relationship between the bed and the air outlet, the controller cannot identify airflow blockage or effective cooling coverage. Consequently, inappropriate louver and fan adjustments frequently amplify temperature overshoot and steady-state oscillation.

To address this fundamental limitation, two core technical challenges are formulated:

1. **Non-intrusive bed localization**: Accurately estimate the horizontal radial distance *d* and azimuth angle θ from the radar projection point to the bed center using low-cost, unmodified hardware. Robust bed clustering is required to eliminate interference from sparse noisy point clouds, human movements and multipath reflections.
2. **Spatially aware steady-state control**: Dynamically adjust louver angles and fan speed based on estimated bed coordinates, real-time return air temperature, and compressor operating parameters. The goal is to suppress $T_{1}$ steady-state fluctuation below the level of traditional variable-frequency systems, while ensuring stable generalization across diverse bedroom layouts without scene-specific training.

This work addresses the above challenges by developing a millimeter-wave radar bed localization algorithm and a cloud-data-trained LGBM weak teacher model. The proposed framework requires no hardware modification and operates fully automatically. Its effectiveness in suppressing temperature oscillation is validated on 719 field-deployed devices.

## 3. Methods

This study aims to identify the bed position using indoor temperature $T_{1}$ from built-in sensors and human activity tracking via millimeter-wave radar, obtaining horizontal radial distance and horizontal azimuth angle of the bed center relative to the ground projection point of the radar. Based on multi-source sensing data, the system dynamically regulates air speed and direction to optimize airflow distribution toward the bed, eliminate interference from bed obstruction, and suppress periodic fluctuations of $T_{1}$ after setpoint attainment. To validate the strategy, a LGBM weak teacher model is constructed using massive cloud-measured datasets, and control parameters are optimized iteratively to minimize $T_{1}$ fluctuation, providing a technical solution for precise bedroom temperature control.

### 3.1. Bed Region Localization Algorithm Based on Millimeter-Wave Point Cloud Clustering

This study adopts a low-cost consumer-grade 1T2R millimeter-wave radar module (ICLegend Micro, Nanjing, China), which is embedded inside commercial wall-mounted air conditioners (Wahin Ultra series, Foshan, China). Different from costly MIMO radar systems, the adopted non-MIMO structure ensures low hardware cost and facilitates large-scale commercial deployment. The detailed radar configuration and hardware parameters are presented below.

RF and Waveform Configuration: The sensor operates at a center frequency of $f_{0}=24\;\mathrm{GHz}$ with a linear frequency-modulated continuous wave (LFMCW) waveform. The sweeping bandwidth is B=185 MHz (24.060–24.245 GHz), and the single chirp duration is $T_{c}=777\;\mu s$. Intermediate frequency (IF) sampling is performed only during the up-chirp phase at a sampling rate of $f_{s}=10.4\;\mathrm{MHz}$.

Temporal and Frame Constraints: The radar works at a fixed frame rate of 20 Hz, corresponding to a frame period of 50 ms. The LFMCW signal adopts an asymmetric sawtooth waveform, and each frame cycle is divided into three stages: (1) Active CPI Phase (24.864 ms): The coherent processing interval contains 32 consecutive chirps. Given $T_{c}=777\;\mu s$ per chirp, the valid radio frequency transmitting and sampling duration occupies 24.864 ms. (2) Data Processing Phase (7 ms): After CPI sampling, the onboard MCU executes FFT operations to extract target range and angle information within a dedicated 7 ms processing window. (3) Sleep Phase (18.136 ms): The system enters a low-power sleep mode for the remaining time to reduce power consumption before the next frame.

**Figure 2:**
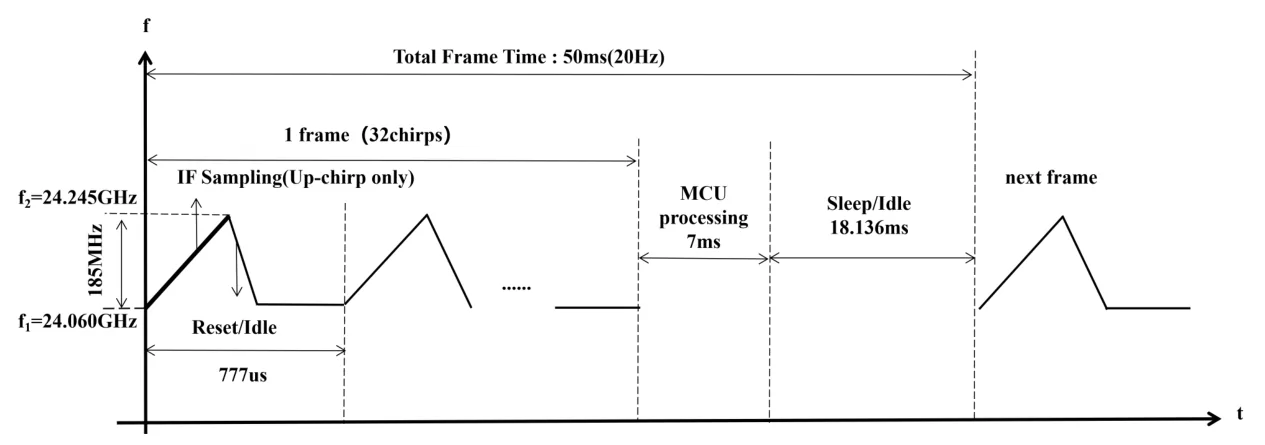
Time-frequency schematic of the LFMCW radar signal operating at a 20 Hz frame rate (50 ms per frame). The radar transmits asymmetric sawtooth waveforms, and each coherent processing interval consists of 32 consecutive chirps with a bandwidth of B=185 MHz ($f_{1}=24.060\;\mathrm{GHz}$ to $f_{2}=24.245\;\mathrm{GHz}$) and chirp duration $T_{c}=777\;\mu s$. IF sampling is conducted only in the up-chirp stage. The valid CPI duration is 24.864 ms, followed by a 7 ms MCU processing window for FFT-based range and azimuth calculation. The system finally enters an 18.136 ms low-power sleep phase to improve energy efficiency.

Figure 2 illustrates the complete time-frequency waveform structure of the adopted radar system.

Physically Deduced Resolution Limits: According to radar electromagnetic theory, the theoretical range resolution is calculated as: ΔR=c/2B≈0.81 m, which satisfies the spatial resolution requirement for indoor bed localization.

Raw radar point clouds contain massive background noise, outliers, and invalid points. Therefore, multi-step data preprocessing is implemented to obtain valid bed-related point cloud features [22,23].

#### 3.1.1. Radar Point Cloud Data Preprocessing

Millimeter-wave radar generates large volumes of raw observation data containing noise, outliers, and redundant points irrelevant to bed localization [22]. The following three preprocessing steps are applied sequentially [23].

(1) Time Window Selection

During nighttime sleep (20:00–08:00), occupants remain recumbent for long periods, producing spatially concentrated point clouds with high signal-to-noise ratio. Only records within this window are retained to suppress interference from daytime activities.

(2) Outlier Removal

Two filtering strategies are implemented for raw radar point cloud preprocessing. First, any data record with missing horizontal radial distance $d_{t}$ of the radar’s ground projection point or horizontal azimuth angle $\theta_{t}$ of the target point at timestamp *t* is discarded. Second, points whose distance exceeds the maximum effective detection limit $d_{\max}=5\;m$ are eliminated. After screening, only point cloud data collected within the sleeping window (20:00–08:00) are reserved to construct the nighttime candidate point set $P_{\mathrm{bed}}$, where each element $z_{t}$ in the set is defined by the paired spatial parameters $\left(d_{t},\theta_{t}\right)$.

(3) Coordinate Transformation and Geometric Compensation

**Figure 3:**
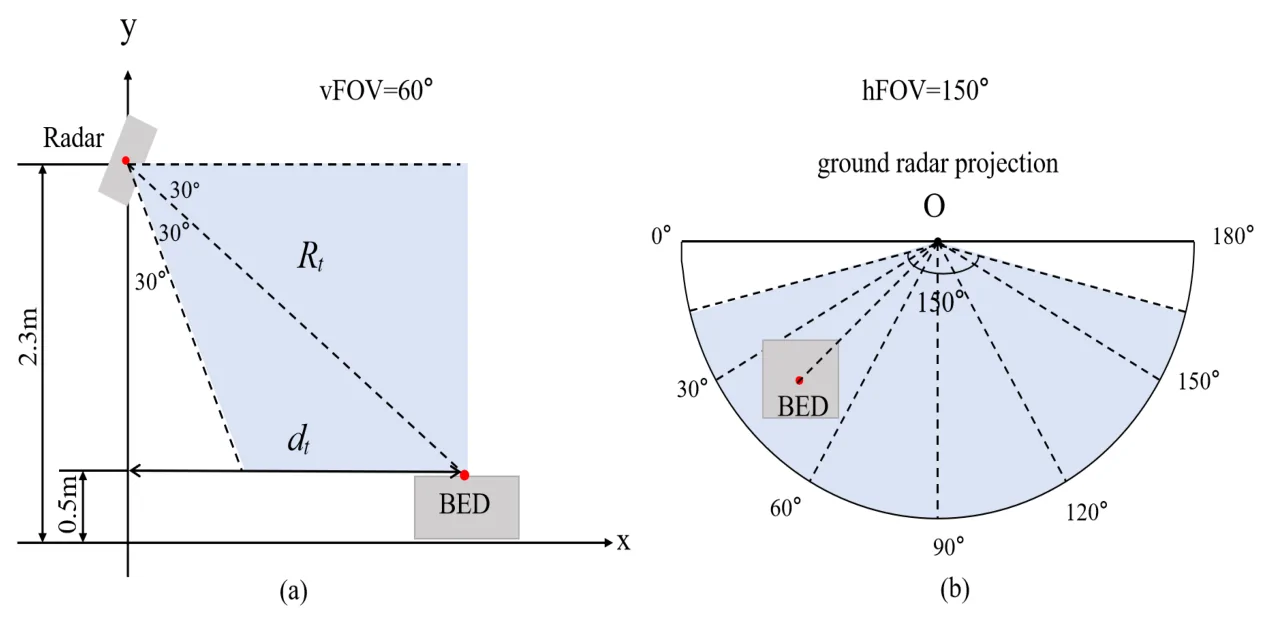
Geometric and Coordinate Projection Model for Spatial Sensing of Millimeter-Wave Radar. (**a**) Side view, illustrating the vertical field of view (vFOV), mounting tilt angle of the radar, as well as the geometric compensation relationship between the radar slant range $R_{t}$ and ground projection distance $d_{t}$. (**b**) Top view, depicting the horizontal field of view (hFOV) and the polar coordinate spatial distribution constructed with the vertical ground projection point *O* of the radar as the origin.

The system establishes a two-dimensional Cartesian coordinate system with the vertical ground projection point of the radar as the origin O(0,0). As illustrated in Figure 3, the fixed installation height of the radar is $H_{\mathrm{radar}}=2.3\;m$, and the standard bed surface height is set to $H_{\mathrm{bed}}=0.5\;m$. The vertical field of view (vFOV) and horizontal field of view (hFOV) of the radar adopted in this paper are 60° and 150°, respectively. To minimize the vertical field-of-view blind zone directly beneath the air conditioner, the radar is mounted with a 30° tilt in the vertical direction.

The azimuth angle solved via radar FFT is denoted as $\theta_{t}$. The range result obtained from radar FFT calculation corresponds to the slant range $R_{t}$ from the radar to the target, and the ground projection distance $d_{t}$ can be calculated by the following formula:

(1) $$d_{t}=\sqrt{R_{t}^{2}-{\left(H_{\mathrm{radar}}-H_{\mathrm{bed}}\right)}^{2}}$$

Finally, the information uploaded from the radar terminal to the cloud consists of the radar ground projection distance and horizontal azimuth angle $\left(d_{t},\theta_{t}\right)$. The uploaded two-dimensional polar coordinates $\left(d_{t},\theta_{t}\right)$ are subsequently converted into two-dimensional Cartesian coordinates $\left(x_{i},y_{i}\right)$ in the follow-up calculation to facilitate spatial clustering based on Euclidean distance [24].

The following four key parameters are determined according to residential bedroom characteristics, hardware specifications, and computational efficiency requirements, and the selection rationality is explained as follows:

1. **Nighttime window (20:00–08:00):** This time period corresponds to residents’ main sleeping hours. During this period, occupants keep static for a long time, and radar point clouds are less disturbed by frequent human activities, which guarantees a high signal-to-noise ratio.
2. **Maximum detection range (5 m):** It matches the effective detection limit of the adopted millimeter-wave radar and fits the depth range of most ordinary residential bedrooms.
3. **Side length of search window (2 m):** This value is designed according to the physical size of mainstream single and double beds, which can completely cover the target bed area.
4. **Sliding step (0.5 m):** A moderate step balances spatial convergence stability and computational cost. A smaller step will greatly increase calculation load, while a larger step will reduce localization resolution.

#### 3.1.2. Behavioral Anchoring-Based Spatial Density Clustering and Bed Position Estimation

The physical estimation of the bed’s center coordinates employs an unsupervised spatial density identification paradigm anchored in human behavior, rather than active radar imaging of the static bed structure. Commercial wall-mounted air conditioners (e.g., the Wahin Ultra series, Foshan, China) are strictly constrained by low-cost, standard hardware configurations, utilizing a conventional one-transmit, two-receive (1T2R) millimeter-wave (mmWave) radar module.

Since a 1T2R radar lacks the physical capability to image a static bed frame, direct detection of the bed is not feasible. Instead, we propose a “human behavior-anchored spatial mapping paradigm.” During the nocturnal sleep window (20:00–08:00), the human body typically remains stationary on the bed for extended periods. Micro-Doppler point clouds triggered by respiration and postural adjustments are highly concentrated spatially and exhibit the highest signal-to-noise ratio (SNR). Consequently, the algorithm isolates these nocturnal human activity point clouds to serve as a priori candidates for the bed’s location. By analyzing the spatial aggregation of these points, the physical boundaries of the bed can be indirectly mapped.

To formalize this behavior-tracking framework and eliminate spatial outliers, this study implements a three-stage spatial density boundary clustering pipeline.

First, the limitations of direct mean estimation are analyzed, demonstrating that simple coordinate averaging is vulnerable to out-of-bed events and noise points, thereby necessitating spatial clustering. Second, the principle of the density-based clustering algorithm is described, focusing on how a sliding window search identifies the cluster with maximum point density. Third, the final bed center is determined as the mean of points within the optimal cluster and then transformed back to polar coordinates $\left(d_{\mathrm{bed}},\theta_{\mathrm{bed}}\right)$, which provides essential spatial inputs for the subsequent LGBM control model.

##### Limitations of Direct Mean Estimation

Although preprocessed candidate points are generally valid, direct coordinate averaging is biased by occasional out-of-bed events (e.g., getting up at night) and noise points from multi-path reflection and clutter [25]. Such outliers shift the global mean, potentially causing localization errors larger than the bed width and leading to incorrect air direction control. Spatial clustering is therefore necessary to separate true bed points from noise and outliers.

##### Principle of Density-Based Clustering

This study adopts a density-based spatial clustering approach to process $P_{bed}$. The core idea is that the bed corresponds to the densest and most concentrated spatial cluster in the point cloud [26].

First, the initial centroid $\left({\bar{x}}_{0},{\bar{y}}_{0}\right)$ of all candidate points is calculated. An initial rectangular search window $W_{0}$ with side length L=2 m (matching typical bed dimensions) is centered at the centroid:

(2) $$W_{0}=\left\{\left(x,y\right)\;\;|x-{\bar{x}}_{0}|\leq \frac{L}{2},\;\left|y-{\bar{y}}_{0}\right|\leq \frac{L}{2}\right\}\;$$

where, $W_{0}$ is initial rectangular search window, L=2 m is side length, and $\left({\bar{x}}_{0},{\bar{y}}_{0}\right)$ is the initial centroid of all candidate points.

A rectangular search window with a side length of 2 m is centered at the initial centroid of all candidate points. The window is shifted 0.5 m in four directions to generate candidate regions, and the window with the highest point density is selected as the core cluster area corresponding to the bed. If multiple windows have equal density, the one closest to the global centroid is selected to ensure stability [27]. The optimal cluster $C_{bed}$ contains points within the best window.

##### Bed Position Identification and Output

The estimated bed center $\left({\hat{x}}_{bed},\;{\hat{y}}_{bed}\right)$ is the mean coordinate of points in $C_{bed}$. The center is converted back to polar coordinates to obtain the horizontal radial distance $d_{bed}$ and horizontal azimuth $\theta_{bed}$ of the bed center relative to the radar’s ground projection point:

(3) $$d_{bed}=\sqrt{{\hat{x}}_{bed}^{2}+{\hat{y}}_{bed}^{2}}$$

(4) $$\theta_{bed}=\arctan2\;\left({\hat{x}}_{bed},\;{\hat{y}}_{bed}\right)$$

where $d_{bed}$ is the calculated horizontal radial distance of the bed center relative to the radar’s ground projection point, and $\theta_{bed}$ is the horizontal azimuth of the bed center relative to the radar’s ground projection point. The output $\left(d_{\mathrm{bed}},\theta_{\mathrm{bed}}\right)$ serves as a key input for the LGBM control model to guide precise air direction and speed regulation.

#### 3.1.3. Statistical Analysis and Evaluation Metrics for Distance/Angle Data

To ensure the mathematical and engineering clarity of the system evaluation framework, a clear conceptual distinction must be established between “Localization Consistency” for broad-scale fields and “Absolute Localization Accuracy” for controlled references.

(1) **Large-Scale Field Localization Consistency**

For large-scale unsupervised cloud measurements encompassing K=719 remote consumer homes, obtaining the physical ground-truth reference coordinates of individual residential bedrooms is unfeasible due to data privacy restrictions and data packet telemetry limitations. Consequently, the spatial aggregation degree and sequence stability of the resolved bed centers over time are quantified via the Minimum Enclosing Circle (MEC) radius, denoted as $R_{\mathrm{MEC}}$, which is calculated utilizing Welzl’s algorithm [28].

Following the American Society of Heating, Refrigerating and Air-Conditioning Engineers (ASHRAE) residential thermal comfort guidelines and standard domestic bed scales [29], an engineering error tolerance threshold of τ=0.5 m is established. For each device j, we judge its localization stability by the radius $R_{MEC,j}$ of the minimum enclosing circle fitted from all estimated bed center coordinates collected on this device. If $R_{MEC,j}$ < 0.5 m, the localization outputs of this device are regarded as spatially stable; otherwise, the positioning results are deemed unstable. This binary judgment rule is further utilized to calculate the overall localization consistency rate across all tested devices.

Thus, the macro-scale system localization consistency rate, denoted as Con(τ), represents the cumulative proportion of devices that achieve secure spatial convergence within the residential boundary:

(5) $$Con\left(\tau \right)=\frac{1}{K}\sum_{j=1}^{K}1_{j}\left(\tau \right)=\frac{\left|\{j|R_{MEC,j}<\tau \}\right|}{K}$$

where Con(τ) corresponds to the total system-wide localization consistency rate across the broad-scale deployment, *K* represents the total number of cloud field devices (K=719), and *j* is the operational device index.

The cumulative distribution function (CDF) of $R_{\mathrm{MEC}}$ across all test devices is adopted to reflect the proportion of devices with a positioning convergence radius smaller than a given threshold *r*. This cumulative proportion is equivalent to the localization consistency rate Con(τ) when the threshold equals τ. We further analyze the mean convergence radius and linear correlation between $R_{\mathrm{MEC}}$ and valid point quantity.

(2) Controlled Absolute Localization Accuracy

The metric Con(τ) defined above characterizes spatial aggregation stability rather than absolute physical displacement from the actual bed geometric center. To bridge this evaluation gap, the “Absolute Localization Accuracy” is reserved strictly for hardware-in-the-loop controlled benchmark environments where the physical spatial ground truth $\left(d_{gt},\theta_{gt}\right)$ can be explicitly measured using a high-precision positioning mat. As elaborated in Section 4.1.1, the absolute physical positioning error within the 2D Cartesian plane ($E_{abs}$) is directly calculated via the polar coordinate outputs by applying the Law of Cosines to secure absolute physical benchmarking verification:

(6) $$E_{abs}=\sqrt{d_{bed}^{2}+d_{gt}^{2}-2d_{bed}d_{gt}\cos\left(\theta_{bed}-\theta_{gt}\right)}$$

where $E_{abs}$ is the isotropic absolute physical positioning error in the 2D Cartesian plane, $d_{bed}$ and $\theta_{bed}$ represent the algorithmically resolved horizontal radial distance and horizontal azimuth of the estimated bed center relative to the radar’s ground projection point, respectively, and $d_{gt}$ and $\theta_{gt}$ correspond to the true horizontal radial distance and true horizontal azimuth benchmark measurements mapped as the physical ground truth.

### 3.2. Control Algorithm Principle

A lightweight LGBM weak teacher model is constructed to address the difficulty of suppressing average indoor temperature fluctuations after setpoint attainment using conventional control. By fusing bed position and air conditioning operational features, the model learns optimal air direction and speed regulation to minimize steady-state $T_{1}$ fluctuations and enhance thermal comfort.

LGBM is selected for the following reasons: Efficient histogram-based decision tree construction and Gradient-based One-Side Sampling (GOSS) reduce memory usage and accelerate training for large-scale cloud data; Support for multi-task learning enables simultaneous prediction of air direction and speed with good interpretability; obustness to heterogeneous features (distance, angle, temperature) matches multi-source fusion requirements.

#### 3.2.1. Light Gradient Boosting Machine (LGBM)

LGBM improves upon Gradient Boosting Decision Tree (GBDT) with a histogram algorithm and GOSS [28,29], greatly enhancing training efficiency for large datasets [30]. The proposed control model is built upon the gradient boosting decision tree framework. Histogram-based optimization and gradient-based one-side sampling are adopted to improve training efficiency for massive cloud datasets [31,32,33].

#### 3.2.2. Definition of the Weak Teacher Model

This paper adopts a multi-task LGBM framework to build a weak teacher model for air conditioner adaptive temperature control. The detailed definition, training label source, training objective and differences from standard LGBM are clarified as follows:

1. **Meaning of the “weak teacher”:** The term “weak” describes the functional positioning of the model rather than poor performance. This model acts as a basic benchmark model, which first learns the conventional control logic of commercial air conditioners from massive real operating data, and serves as the base for further optimization. It is not designed as a standalone high-performance optimal controller.
2. **Source of training labels:** All supervision labels for louver angles and fan speed are extracted from historical real control commands of 719 physical air conditioners stored in the cloud platform. These records include automatic adjustment actions of the original on-board controller and stable operation data under daily user scenarios.
3. **Training objective:** The core training objective is to suppress the steady-state fluctuation of return air temperature caused by bed obstacles and human heat sources, while maintaining reasonable and stable air supply control.
4. **Differences from standard LGBM classifiers:** A standard LGBM usually performs single classification or regression tasks. In contrast, our model adopts a multi-task learning architecture, which simultaneously predicts the states of larger louver, small louver and fan speed. Besides common classification loss, we further introduce temperature fluctuation loss and consistency regularization to constrain model output, which is a customized improvement for air conditioning control scenarios.
5. **Explanation on model output:** High classification accuracy indicates that the model fully learns the effective control rules of the original controller. However, this work does not aim to simply imitate existing control strategies. By fusing millimeter-wave radar bed position information and adopting a fluctuation-oriented loss function, the model re-optimizes air direction and fan speed, so as to achieve better temperature stability and thermal comfort than the original controller.

#### 3.2.3. LGBM Model for Air Conditioning Control

The model is trained on approximately 105,000 cloud records, including radar data, indoor temperature, setpoint temperature, compressor frequency, and return air temperature $T_{1}$. It preserves user setpoints and only optimizes air direction and speed to stabilize $T_{1}$.

##### Input Feature Space

The model takes five-dimensional features:

(7) $$X=\{d,\theta ,T_{1},f,△T_{set}\}$$

where *d* is the horizontal radial distance of the bed center relative to the radar’s ground projection point (0–5 m), θ is the horizontal azimuth (30°–150°), $T_{1}$ is real-time return air temperature (°C), *f* is compressor frequency (0–100), and $△T_{set}=\left|T_{1}-T_{set}^{raw}\right|$ is temperature deviation (°C); $△T_{set}<0.5$ °C indicates setpoint attainment.

Features are standardized to eliminate scale effects.

##### Output Space

Outputs preserve $T_{set}$ and predict control parameters:

(8) $$Y=\left\{T_{set},D_{big},D_{small},V\right\}$$

where, $D_{big}$ is left/right large louver (binary), $D_{small}$ is left/right small louver (binary), and *V* is fan speed (6 classes: 1–5 fixed, 6 auto). The above control labels are derived from cloud historical control records of real devices.

##### Multi-Task Loss Function

A weighted loss prioritizes $T_{1}$ fluctuation minimization:

(9) $$L_{weak}=\omega_{fluc}L_{fluc}+(1-\omega_{fluc})(\omega_{big}L_{cls}^{big}+\omega_{small}L_{cls}^{small}+\omega_{wind}L_{cls}^{wind})$$

where, $\omega_{fluc}=0.7$ is weight of fluctuation loss, $L_{fluc}$ is fluctuation loss, $\omega_{big}=0.4$ is weight of large louver classification loss, $L_{cls}^{big}$ is large louver classification loss, $\omega_{small}=0.3$ is weight of small louver classification loss, $L_{cls}^{small}$ is small louver classification loss, $\omega_{wind}=0.3$ is weight of fan speed classification loss, $L_{cls}^{wind}$ is fan speed classification loss.

The core fluctuation loss is:

(10) $$L_{fluc}=\frac{1}{N_{reach}}\sum_{i=1}^{N_{reach}}\left|T_{1,i}-T_{set,i}^{raw}\right|$$

where, $L_{fluc}$ is core fluctuation loss, $N_{reach}$ is number of samples reaching setpoint, $T_{1,i}$ is return air temperature of sample *i*, and $T_{set,i}^{raw}$ is original set temperature of sample *i*. Classification losses use cross-entropy.

##### Self-Supervised Consistency Regularization

To improve robustness to feature noise, consistency regularization is added. Consistency regularization consists of two constraints, namely feature consistency and temperature fluctuation consistency, which suppress output jitter induced by noise from millimeter-wave radar point clouds.

Final loss:

(11) $$L_{weak}^{final}=L_{weak}+\lambda_{consist}L_{consist}$$

where $\lambda_{consist}=0.1$ is the regularization weight, used to balance the objectives of minimizing fluctuations and optimizing model robustness. The above joint loss function is optimized following the implementation rules and weight configuration presented in Section 3.2.4.

#### 3.2.4. Loss Function Implementation and Weight Configuration

##### Optimization of Loss Terms in LGBM Model

The proposed multi-task learning framework integrates temperature fluctuation loss, classification loss, and consistency regularization, which are jointly optimized throughout the gradient boosting iterations of the LGBM model. Detailed implementation mechanisms are described as follows.

The temperature fluctuation loss is defined as the primary optimization objective. During the training process, gradients and second-order derivatives are calculated based on the absolute deviation between the real-time return air temperature and the user-set temperature. These derivatives guide the splitting of decision tree nodes and the assignment of leaf node values, so as to continuously suppress the steady-state temperature fluctuation of the air conditioning system. This loss term acts on all valid samples collected after the temperature reaches the set value, prioritizing the stability of indoor temperature.

The classification loss adopts cross-entropy function for the prediction of louver states and fan speed. It serves as an auxiliary constraint to ensure that the predicted control commands conform to the operational logic of practical air conditioners and avoid unreasonable output during model inference.

The consistency regularization is incorporated into the overall loss function as a penalty term. In each iteration of tree training, it imposes constraints on both input features and model outputs. This design alleviates the adverse effects of noise from millimeter-wave radar point clouds and random environmental disturbances, and further enhances the robustness of the entire control model. All loss components participate in parameter updating via back-propagation in each boosting round.

In summary, the LGBM model takes the minimization of total joint loss as the global optimization target. Temperature stability, control rationality, and output consistency are comprehensively optimized in the hierarchical construction of decision trees.

##### Selection and Sensitivity Analysis of Loss Weights

The weights of all loss components, including the fluctuation loss weight $\omega_{fluc}$, classification loss weights, and consistency regularization weight $\lambda_{consist}$, are determined through systematic parameter screening and sensitivity analysis.

1. **Parameter selection procedure:** We first define a reasonable value range for each weight parameter. A grid search strategy is adopted to traverse different weight combinations within the preset ranges. All candidate combinations are tested on the same training and validation datasets. The final weight configuration is selected by comprehensively evaluating three core indicators: return air temperature fluctuation, classification accuracy of control components, and model generalization ability. The optimal combination is determined as $\omega_{fluc}=0.7$, $\omega_{big}=0.4$, $\omega_{small}=0.3$, $\omega_{wind}=0.3$, and $\lambda_{consist}=0.1$. This configuration guarantees excellent fluctuation suppression performance while maintaining high accuracy of louver and fan speed control.
2. **Sensitivity analysis of core weights:** We conduct sensitivity tests on key weights to verify model robustness. When $\omega_{fluc}$ varies within the range of [0.5, 0.9], a larger weight leads to better temperature stability but slightly reduces the classification accuracy of fan speed. For the consistency regularization weight $\lambda_{consist}$ ranging from 0 to 0.2, an excessively small value will cause unstable model outputs, while an overly large value will weaken the model’s fitting capability to real operating data. The adopted weight values lie in the optimal interval with balanced overall performance.
3. **Practical empirical justification:** From the perspective of practical application in bedroom scenarios, human thermal comfort is far more sensitive to temperature fluctuation than minor deviations of air supply gears. Therefore, we assign a higher weight to temperature fluctuation loss, which is consistent with the actual usage demands of residential air conditioners.

##### Core Technical Innovations of the Control Model

The technical contributions of the proposed framework are summarized as follows:

1. **Weak teacher prior supervision:** A weak teacher model is built on original air conditioner operation data to provide effective prior knowledge. It compensates for the scarcity of optimal control labels in bedroom scenarios and guides stable model optimization.
2. **Multi-task joint optimization:** A weighted dual-task loss is designed to simultaneously optimize temperature fluctuation suppression and equipment state prediction. This mechanism balances thermal comfort and operational feasibility, outperforming conventional single-task schemes.
3. **Bed-position-based spatial adaptive control (core novelty):** Different from temperature-only control, our method takes radar-sensed bed spatial features as the core input to achieve targeted regional airflow regulation. Consistency regularization plays an auxiliary stabilizing role, and the essential technical innovation lies in the introduced user spatial perception mechanism.

Overall, the combination of weak teacher prior, multi-task optimization, and bed spatial input realizes an innovative and practical spatial-aware intelligent control solution for bedroom air conditioning systems.

#### 3.2.5. Model Training and Hyperparameters

The dataset is split 7:3 for training and validation. The training labels correspond to practical control actions executed by the original air conditioner controller. Early stopping is applied if validation loss does not improve for 10 consecutive iterations. Key hyperparameters are listed in Table 1.

##### Model Evaluation Metrics

Model evaluation metrics are divided into core metrics and auxiliary metrics. Core metrics are about mean absolute fluctuation, variance, and no fluctuation ratio. Auxiliary metrics are about accuracy and $F1_{score}$.

(1) Core metrics
  (a) Mean Absolute Fluctuation (${MAF}_{T_{1}}$):
    (12) $${MAF}_{T_{1}}=\frac{1}{N_{reach}}\sum_{i=1}^{N_{reach}}\left|T_{1,i}-T_{set,i}^{raw}\right|$$
    where ${MAF}_{T_{1}}$ is mean absolute fluctuation of return air temperature; N_reach_ is number of samples reaching setpoint;
  (b) Variance (${VAR}_{T_{1}}$) and No Fluctuation Ratio (${NFR}_{T_{1}}$):
    Apart from the core metric mean absolute fluctuation ${MAF}_{T_{1}}$, we also calculate the variance of return air temperature ${VAR}_{T_{1}}$ and the no-fluctuation ratio ${NFR}_{T_{1}}$, which refers to the proportion of samples satisfying $\left|T_{1,i}-T_{set,i}^{raw}\right|<0.1$ °C, to support the analysis of steady-state control stability.
(2) Auxiliary metrics
  Cross-entropy loss is adopted as the training objective for louver and fan speed classification tasks. After model inference, accuracy and $F1_{score}$ are further calculated as auxiliary metrics to quantify the control prediction performance.

## 4. Results

### 4.1. Radar-Based Bed Target Localization

#### 4.1.1. Laboratory Benchmark Validation and Quantitative Comparison of Clustering Algorithms

To verify the localization accuracy of the proposed bed center identification algorithm, benchmark experiments are carried out in a laboratory equipped with a high-precision positioning mat for distance and angle calibration.

##### Experimental Setup

**Figure 4:**
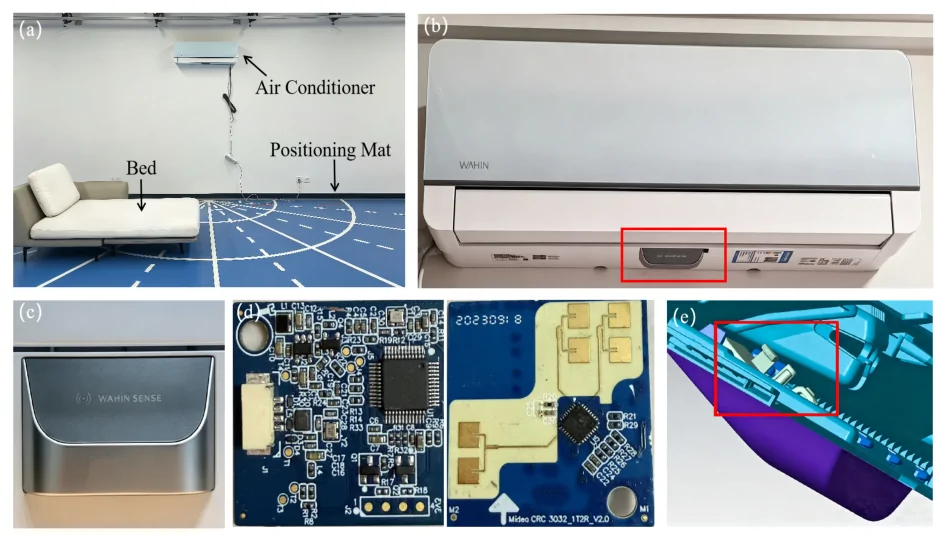
Hardware integration and experimental deployment of the millimeter-wave radar system. (**a**) Photograph of a representative experimental setup. The air conditioner is mounted on the wall at a height of 2.3 m, and the positioning mat is placed directly beneath the indoor unit. The other experimental configurations use the same equipment arrangement, with only the bed position being changed. (**b**) Front view of the air-conditioner indoor unit; the red box highlights the external radar installation area, which is shown at an enlarged scale in panel (**c**). (**c**) Close-up view of the external panel with a wave-transparent cover for the radar sensor. (**d**) Front and rear photographs of the 1T2R millimeter-wave radar module. (**e**) Three-dimensional CAD model of the internal chassis; the red box highlights the internal radar mounting assembly and its fixed 30° downward mounting orientation.

As illustrated in Figure 4, the radar is mounted directly beneath the air conditioner indoor unit to avoid electromagnetic interference from the compressor and peripheral components. The installation height of the air conditioner is fixed at 2.3 m with a downward tilt angle of 30°. Before data collection, the ground-truth polar coordinates $\left(d_{\mathrm{gt}},\theta_{\mathrm{gt}}\right)$ from the radar projection point to the bed geometric center are measured using the positioning mat and adopted as reference values.

**Figure 5:**
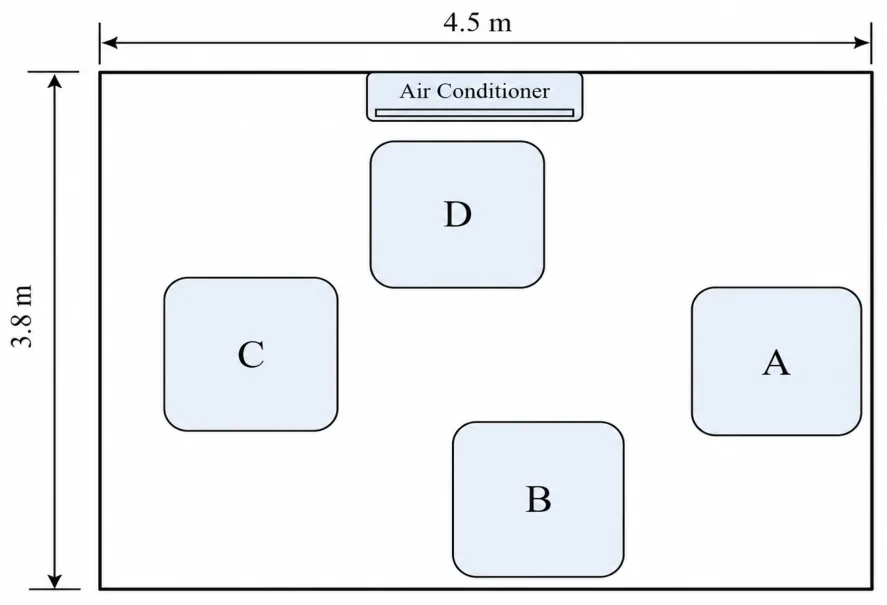
Floor plan of the 4.5 m × 3.8 m laboratory test room. The layout shows the air conditioner installation position and four preset ground-truth bed locations A, B, C, and D.

The radar outputs human trajectory data to the server every 15 s for storage and analysis, while the estimated bed centroid coordinates $\left(d_{n},\theta_{n}\right)$ are updated hourly. A 10 h continuous experiment is designed to mimic real bedroom behavior: each hour consists of 50 min simulated in-bed rest and 10 min random out-of-bed activities. Experiments are performed independently at four representative bed positions (A, B, C, D) to evaluate algorithm robustness under diverse spatial layouts, as shown in Figure 5.

##### Experimental Results

**Figure 6:**
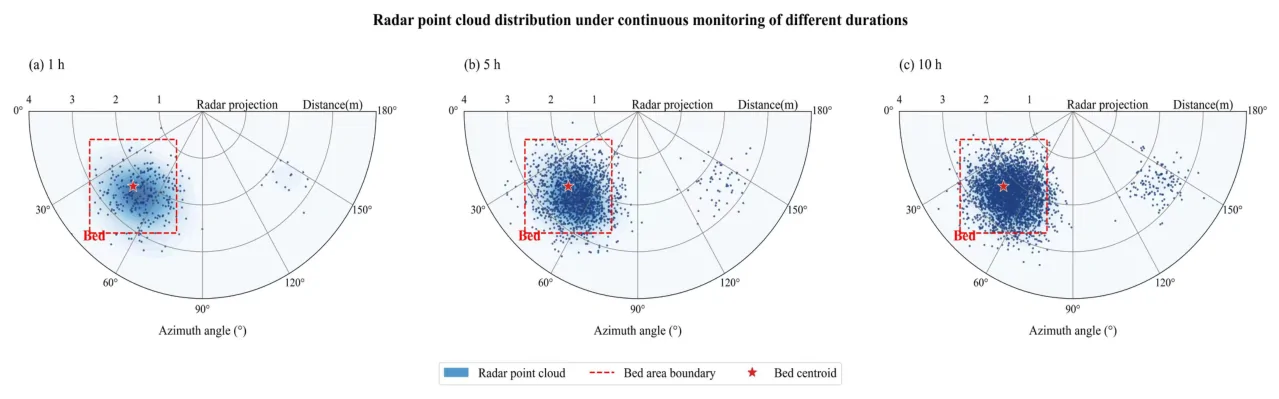
Bed-position recognition results at a representative bed position for cumulative observation windows of (**a**) 1 h, (**b**) 5 h, and (**c**) 10 h. Blue points represent all radar point clouds acquired within each observation window; the red star marks the estimated bed centroid, and the red dashed box outlines the detected bed region.

Figure 6 presents localization results at Position C for cumulative observation windows of 1 h, 5 h, and 10 h. Blue dots denote human activity trajectories, and red stars indicate estimated bed centers. Longer accumulation windows capture richer activity information and suppress random noise, yielding progressively more stable bed center estimation.

To quantify absolute localization accuracy, the bed centroid polar coordinates estimated after 10 h data accumulation are compared against ground truth. Using identical input datasets, the proposed algorithm is further compared with three classic clustering approaches: DBSCAN, K-Means and Mean-Shift. Absolute localization errors for all schemes are summarized in Table 2.

The proposed method achieves an average absolute localization error of only 0.255 m, outperforming all baseline clustering algorithms. DBSCAN (average error: 0.407 m) is sensitive to density hyperparameters, and centroid drift easily occurs under local multipath noise. Without spatial size priors, K-Means (0.449 m) and Mean-Shift (0.439 m) are vulnerable to residual outlier noise outside the bed area, leading to outward centroid bias. The proposed algorithm achieves centimeter-level localization precision while maintaining low computational overhead.

#### 4.1.2. Statistical Results of Field Tests

The localization algorithm is deployed on commercial air conditioner terminals. With user authorization, cloud telemetry data from 719 devices are extracted for large-scale evaluation. Due to privacy constraints, ground-truth bed coordinates and room dimensions cannot be acquired for individual households. Accordingly, the minimum enclosing circle radius $R_{\mathrm{MEC}}$ is adopted to characterize spatial convergence stability of sequential bed center estimations.

**Figure 7:**
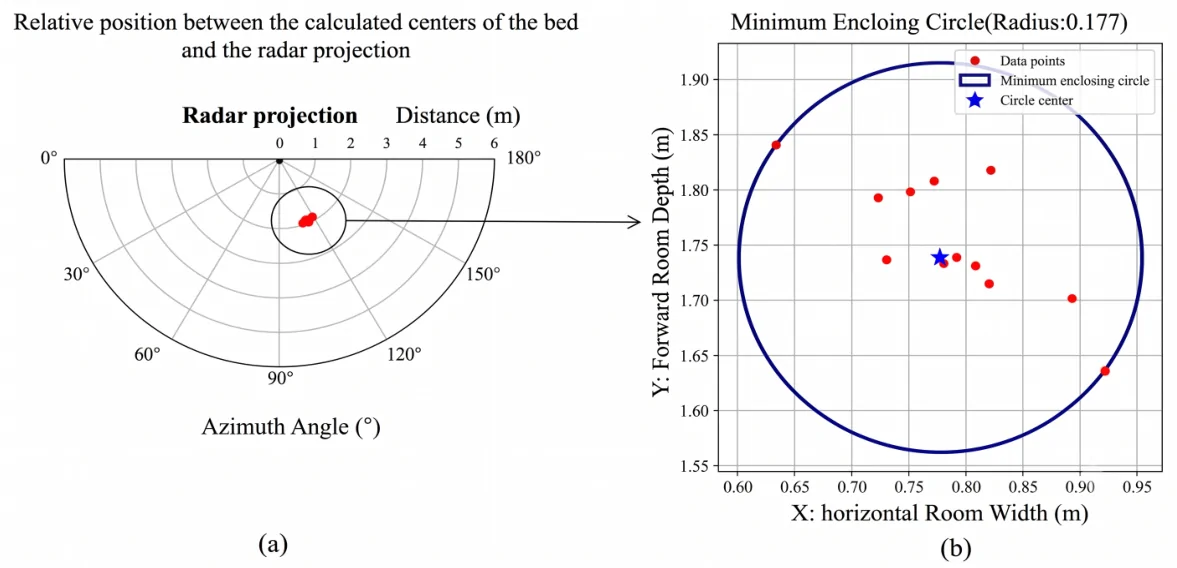
Spatial consistency evaluation of behavior-anchored bed localization based on cloud data from a field-deployed device. (**a**) Relative position between the estimated bed center and radar projection point. (**b**) Minimum enclosing circle fitted from sequential bed center estimations.

Figure 7 illustrates typical behavior-anchored clustering outputs from one field device. The coordinate origin corresponds to the radar ground projection point, and all distances are geometrically compensated ground-plane ranges. The resolved bed center is located at (0.78, 1.74) with a convergent MEC radius $R_{\mathrm{MEC}}=0.177$ m. The scattered red points represent long-term accumulated micro-Doppler point clouds generated by nocturnal body movements, rather than static bed boundary outlines.

All tested units belong to the mass-produced Wahin Ultra variable-frequency wall-mounted air conditioner series. The radar modules are factory-integrated with standardized mounting: 2.3 m height and fixed 30° downward tilt, eliminating hardware bias across devices. The cloud dataset naturally covers diverse real-world conditions: bedroom areas ranging from 10–25 $m^{2}$, varying bed sizes and orientations, and heterogeneous user behaviors including irregular sleep schedules, frequent body movement, and transient out-of-bed events.

**Figure 8:**
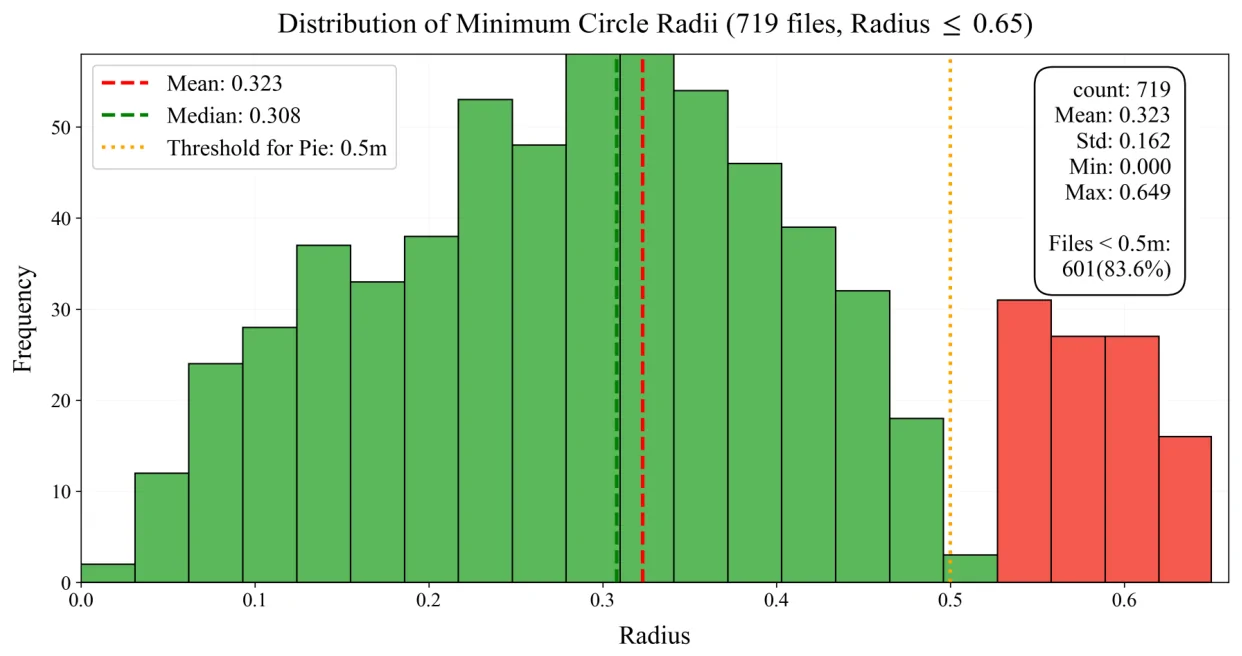
Histogram of minimum enclosing circle radii for all 719 test devices. Most $R_{\mathrm{MEC}}$ values are concentrated within 0.1–0.5 m.

**Figure 9:**
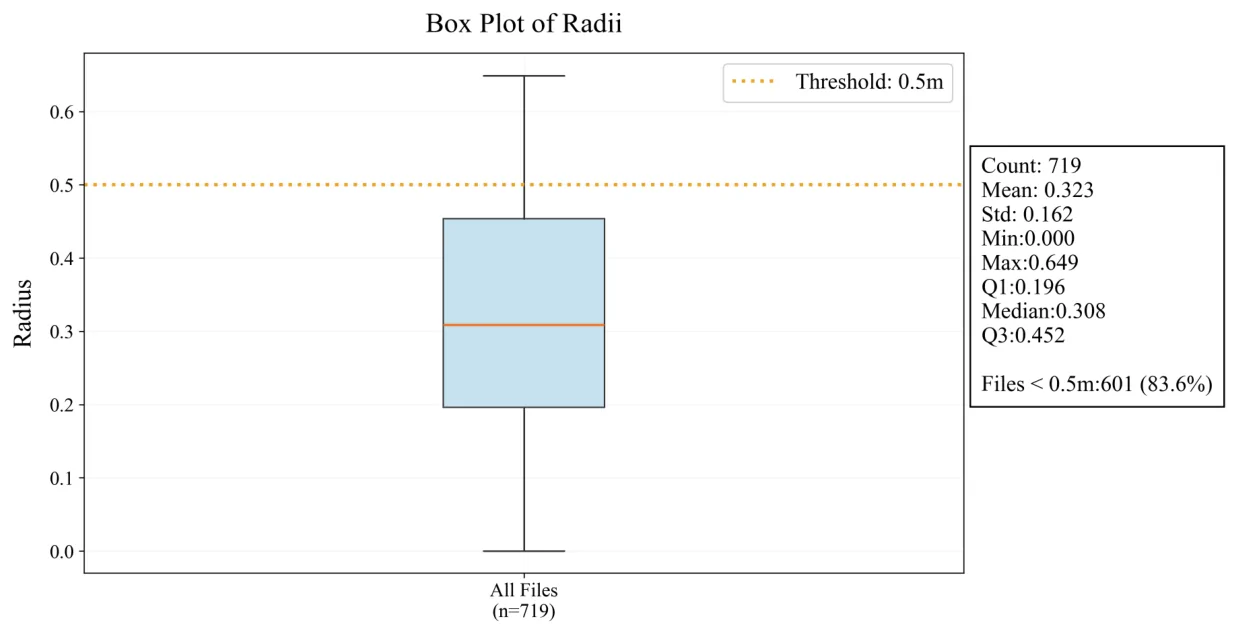
Boxplot of the minimum enclosing circle radii $R_{\mathrm{MEC}}$ for 719 test devices. The light-blue box spans the interquartile range from $Q_{1}=0.196\;m$ to $Q_{3}=0.452\;m$, and the solid orange line inside the box indicates the median of 0.308 m. The orange dotted horizontal line marks the error-tolerance threshold of 0.5 m, below which 601 of the 719 devices (83.6%) are located.

**Figure 10:**
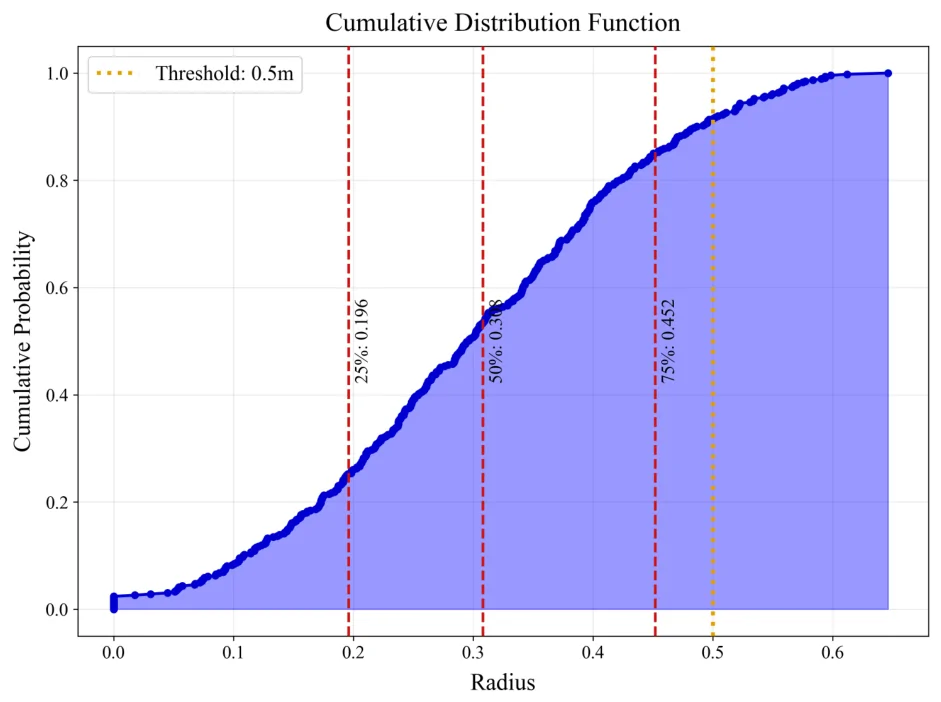
CDF curve of minimum enclosing circle radii, showing the proportion of devices with localization convergence radius below a given threshold.

**Figure 11:**
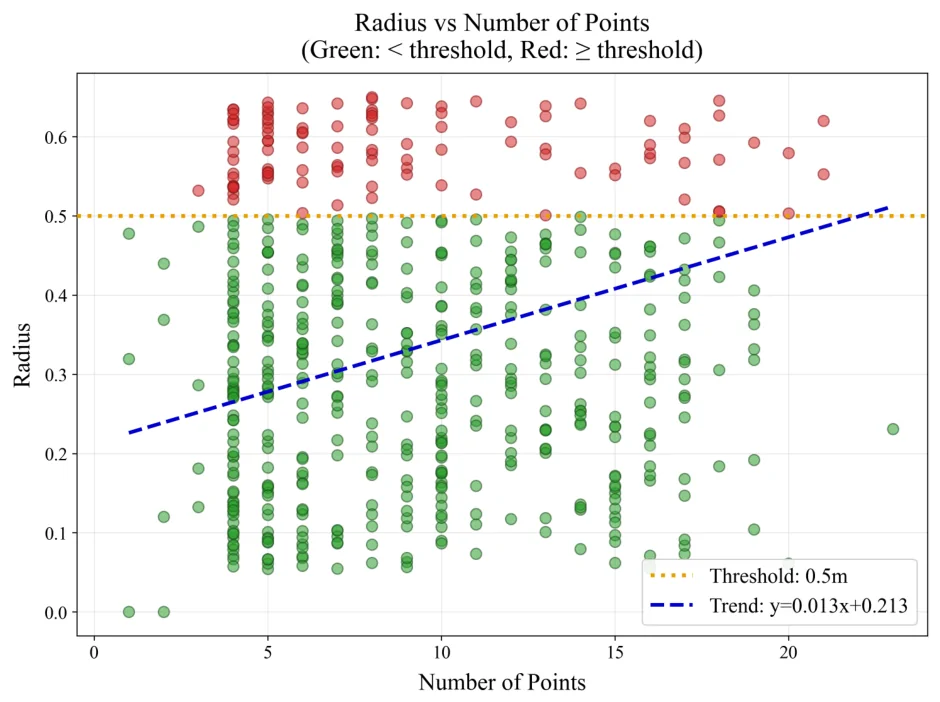
Correlation between the minimum enclosing circle radius $R_{\mathrm{MEC}}$ and the number of valid candidate points *N*. Green dots denote qualified localization results with $R_{\mathrm{MEC}}<0.5\;m$, whereas red dots denote unqualified results with $R_{\mathrm{MEC}}\geq 0.5\;m$. The orange dotted horizontal line indicates the 0.5 m error-tolerance threshold, and the blue dashed line represents the fitted relationship ${\hat{R}}_{\mathrm{MEC}}=0.013N+0.213$, indicating a weak positive correlation. Because *N* is a discrete integer-valued variable, multiple observations share the same horizontal coordinate, resulting in vertically aligned and partially overlapping point clusters.

Statistical analysis demonstrates satisfactory localization stability under diverse interference conditions. As shown in Figure 8, the distribution of $R_{\mathrm{MEC}}$ is unimodal and right-skewed, with most values concentrated within 0.1–0.5 m, a mean of 0.323 m, and a median of 0.308 m. The boxplot in Figure 9 further shows that the interquartile range of $R_{\mathrm{MEC}}$ lies entirely below the error threshold τ=0.5 m. As shown in Figure 10, the cumulative distribution function yields F(0.5)=0.836, indicating that 83.6% of the devices satisfy the 0.5 m consistency criterion under blind real-world deployment. Finally, Figure 11 shows a mild positive linear correlation between $R_{\mathrm{MEC}}$ and the number of valid candidate points, with the fitted relationship ${\hat{R}}_{\mathrm{MEC}}=0.013N+0.213$. Nevertheless, most samples remain within the qualified threshold.

### 4.2. Air Conditioning Control Algorithm Results

#### 4.2.1. Model Convergence Analysis

**Figure 12:**
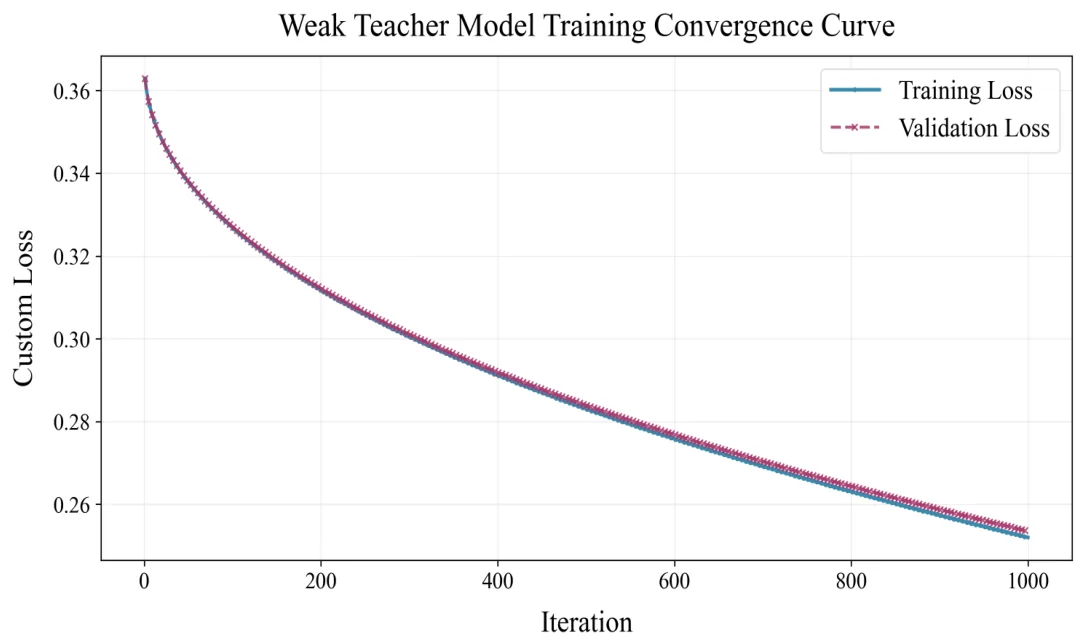
Training and validation loss curves of the LGBM weak teacher model. Loss decreases steadily and converges in the later stage without evident overfitting.

As shown in Figure 12, training and validation loss converge smoothly without obvious overfitting, verifying that the model effectively learns the mapping between input features and control outputs and possesses favorable generalization. Early stopping is adopted to improve computational efficiency.

#### 4.2.2. Feature Importance Analysis

**Figure 13:**
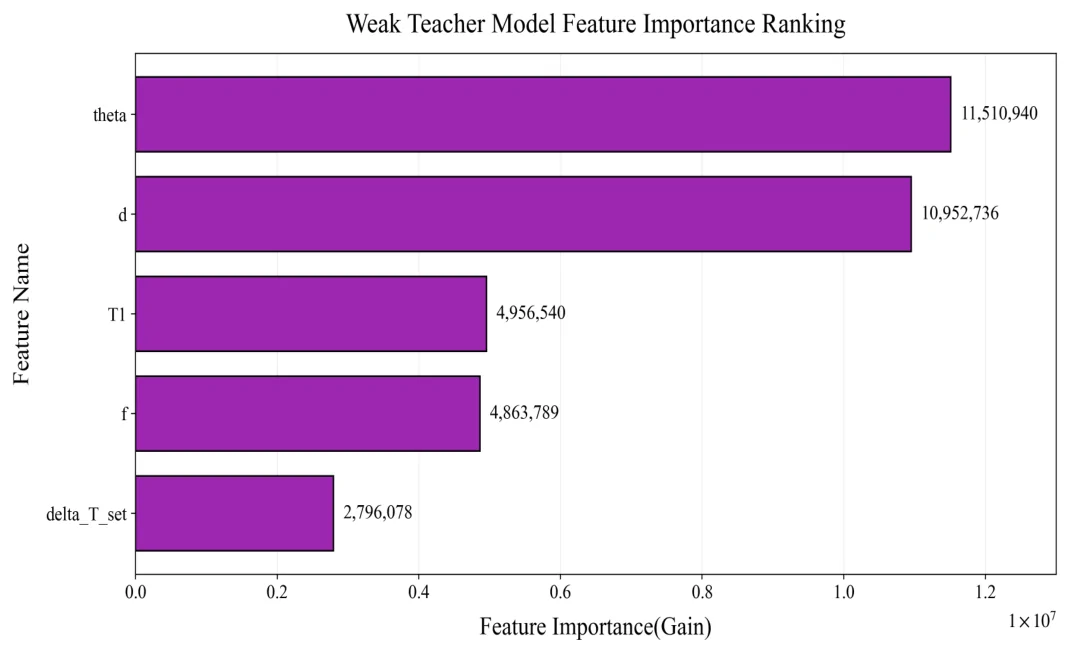
Feature importance ranking of the LGBM control model. Bed horizontal azimuth θ and radial distance *d* rank highest, demonstrating that spatial perception serves as a core factor for airflow optimization and indoor temperature stabilization.

As shown in Figure 13, Feature importance reveals that bed horizontal azimuth θ is the dominant input. It determines the horizontal orientation of the bed relative to the indoor unit and directly guides airflow arrangement to homogenize temperature distribution and suppress steady-state $T_{1}$ oscillation. The radial distance *d* is the second most important feature, governing required air supply intensity: higher fan speed ensures coverage for distant zones, while lower speed avoids local discomfort for nearby regions. Real-time return air temperature $T_{1}$ ranks third, reflecting instantaneous indoor thermal conditions. Temperature deviation $\Delta T_{\mathrm{set}}$ and compressor frequency *f* act as auxiliary features to identify steady-state intervals and quantify system load. The results confirm that stable post-setpoint temperature control relies on accurate bed spatial information combined with real-time thermal feedback.

#### 4.2.3. Core Fluctuation Metric Analysis

**Figure 14:**
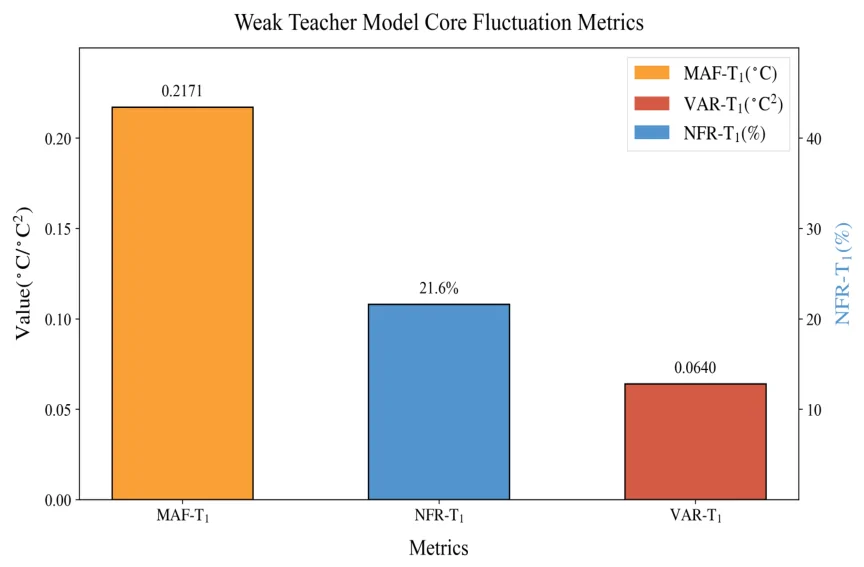
Core steady-state evaluation metrics on the validation set, including mean absolute fluctuation $MAF_{T_{1}}$, temperature variance $VAR_{T_{1}}$ and non-fluctuation ratio $NFR_{T_{1}}$.

On validation samples after reaching target temperature, $MAF_{T_{1}}=0.21$ °C, $VAR_{T_{1}}=0.064\;{}^{\circ}C^{2}$, and $NFR_{T_{1}}=21.6\%$, as shown in Figure 14. The return air temperature $T_{1}$ is stabilized within approximately ±0.2 °C of the setpoint, significantly outperforming conventional fixed-frequency and variable-frequency schemes. The above offline results are derived from historical cloud data of 719 air conditioners. Further closed-loop real-machine tests demonstrate that the mean absolute fluctuation can be reduced to 0.188 °C under fixed bed coordinates. Detailed comparative results with baseline controllers are presented in subsequent subsections.

#### 4.2.4. Auxiliary Metric Analysis

**Figure 15:**
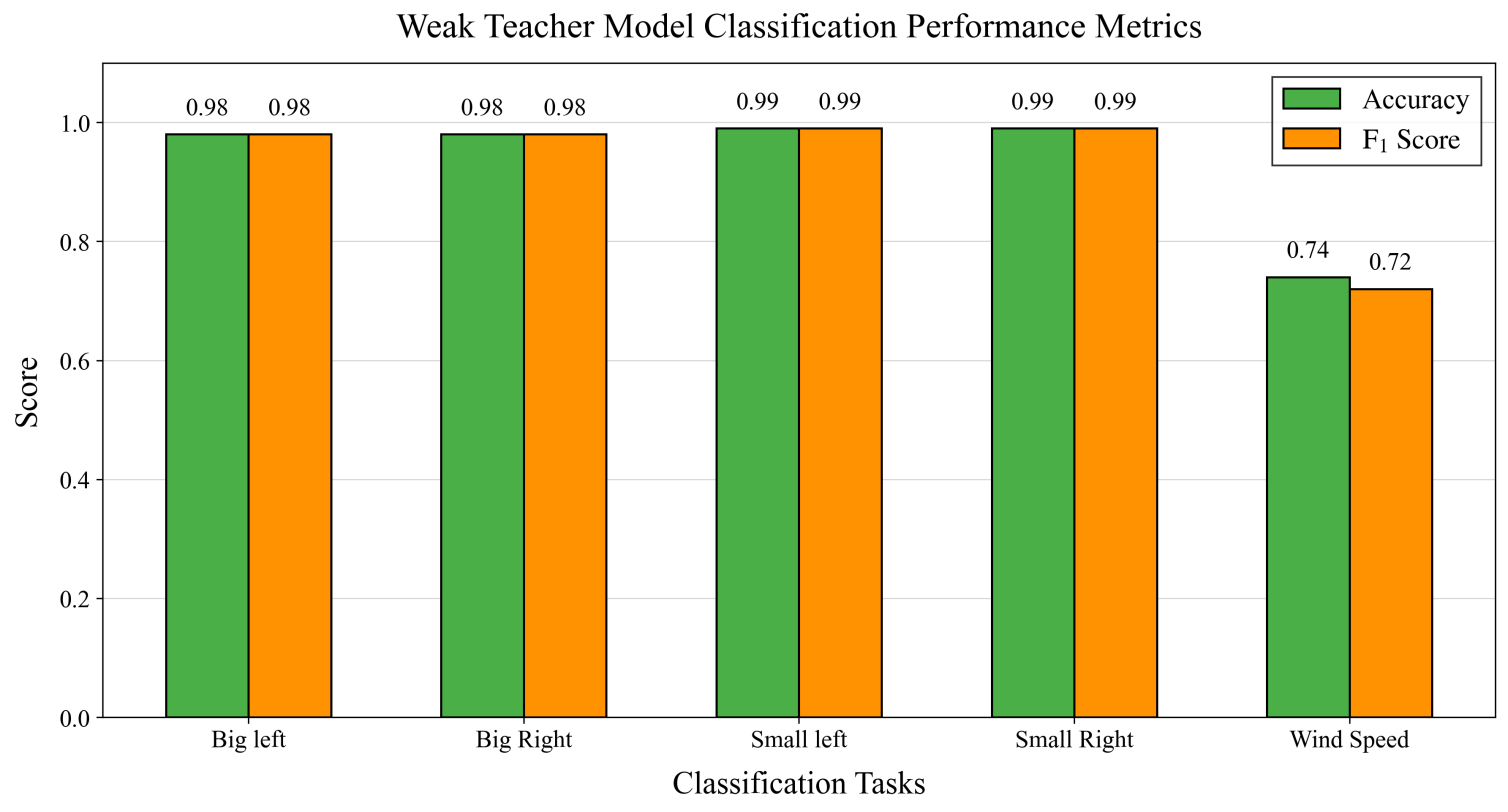
Auxiliary classification metrics for louver and fan speed prediction. Large and small louver classification achieves accuracy and an F1-score above 0.98, while fan speed prediction yields relatively lower metrics.

As shown in Figure 15, the small and large louver control tasks achieve accuracy and F1-score near 0.98, while fan speed classification obtains accuracy of 0.74 and F1-score of 0.72. High precision for louver adjustment guarantees reliable airflow steering. Moderately lower fan speed performance reflects the complexity of multi-level speed classification, yet the model still captures dominant operating patterns for practical deployment.

#### 4.2.5. Ablation Experiments

Ablation tests are carried out under identical experimental conditions to verify the effectiveness of bed spatial features, consistency regularization, and other input variables. Six model variants are compared: full model, model without bed position inputs (no_pos), model without consistency regularization (no_reg), model excluding $T_{1}$, model excluding compressor frequency *f*, and model excluding temperature deviation $\Delta T_{\mathrm{set}}$. Quantitative results are listed in Table 3.

Removing return air temperature $T_{1}$ leads to the worst control performance, confirming that $T_{1}$ acts as the core closed-loop feedback signal. Discarding bed spatial features d,θ causes obvious growth of temperature fluctuation, verifying that millimeter-wave bed localization provides critical information to balance indoor airflow. Excluding compressor frequency *f* or temperature deviation $\Delta T_{\mathrm{set}}$ only induces mild performance degradation, indicating they serve as auxiliary operating-state inputs. Removing consistency regularization substantially amplifies temperature oscillation, which proves the proposed constraint effectively suppresses output jitter originating from radar point cloud noise.

#### 4.2.6. Real Device Comparative Experiment

Online closed-loop comparative tests are implemented on a physical air conditioner in a 4.5 m × 3.8 m laboratory bedroom under cooling mode with set temperature 26 °C, to compare steady-state control performance across multiple schemes. Five control strategies are evaluated:

1. **Traditional PID Control**: Classical temperature closed-loop PID, adopted as the conventional baseline.
2. **Original factory automatic control**: The air conditioner runs under default automatic mode with all airflow parameters self-adjusted.
3. **XGBoost Single-Task Control/Vanilla Single-Task LGBM Control:** Both schemes adopt basic single-task control frameworks without bed position spatial features, multi-task branches, or consistency regularization. The only difference lies in the core learning models, which are XGBoost (version 2.0.3) and LGBM (version 4.3.0), respectively.
4. **Proposed algorithm**: The bed position is fixed at d=3.8 m, $\theta =69^{{}^{\circ}}$. The LGBM controller dynamically adjusts louver angles and fan speed.

Three core indicators are adopted: $MAF_{T_{1}}$, $VAR_{T_{1}}$ and $NFR_{T_{1}}$. Full experimental results are summarized in Table 4.

All baseline schemes exhibit significantly larger temperature fluctuation than the proposed spatially aware multi-task LGBM controller. Traditional PID only relies on single-point temperature feedback with fixed gains; it passively compensates temperature deviation after fluctuation occurs and cannot account for bed-induced airflow distortion and human heat loads. Vanilla single-task gradient boosting models omit bed spatial features and lack consistency regularization, failing to mitigate airflow disturbance from indoor obstacles. The comparison validates that integrating millimeter-wave spatial perception and multi-task consistency constraints is the key to achieving superior steady-state temperature stability.

## 5. Discussion

This work develops a millimeter-wave radar-assisted LGBM weak teacher control algorithm to mitigate steady-state indoor temperature fluctuations of residential air conditioners after temperature setpoint stabilization. Validated through laboratory calibration and large-scale field tests on mass-produced devices, the proposed method yields significant thermal stability improvements over conventional control schemes. Unlike traditional temperature-only closed-loop strategies that rely solely on historical thermal errors, this method integrates spatial perception of indoor sleeping areas to guide adaptive airflow regulation, effectively optimizing indoor thermal environment stability.

The proposed bed localization framework integrates night activity filtering, outlier elimination, density-based clustering, and minimum enclosing circle (MEC) evaluation to achieve robust spatial positioning. Field tests on 719 commercial devices obtain a mean MEC radius of 0.323 m and an 83.6% localization consistency rate within a 0.5 m error tolerance. Laboratory physical calibration further achieves a centimeter-level average absolute localization error of 0.255 m, outperforming conventional clustering algorithms. Accurate bed position estimation provides reliable spatial features, serving as the core foundation for high-precision steady-state temperature regulation.

Feature importance analysis verifies that bed azimuth and radial distance dominate model decision-making, compensating for the lack of spatial awareness in traditional thermostat-based control. Benefiting from multi-task learning and consistency regularization, the proposed system reduces steady-state mean absolute temperature fluctuation and temperature variance to 0.21 °C and 0.064 °C^2^, respectively. Compared with fixed-frequency (±2–3°C) and conventional variable-frequency (≈±1 °C) air conditioners, the proposed method achieves prominent stability gains, with distinct advantages in low-cost deployment, high universality, and unattended operation.

### 5.1. Analysis of Localization Failure Cases

Although the proposed localization algorithm achieves high overall robustness, 16.4% of field devices fail to meet the 0.5 m localization tolerance standard. Combining millimeter-wave radar sensing mechanisms and practical bedroom deployment scenarios, the main causes of localization failure are summarized as follows:

1. **Millimeter-wave multipath interference**: Enclosed and furniture-dense bedrooms induce complex multipath reflections of radar signals. Mixed reflected point clouds interfere with valid bed and human activity clusters, dispersing point cloud distribution and increasing localization errors.
2. **Scene occlusion and irregular layout**: Large obstacles such as cabinets and curtains around the bed block radar detection signals, distorting the spatial distribution of effective point clouds and causing estimation deviation.
3. **Dynamic human and pet interference**: Continuous movement of multiple occupants or pets near the bed generates abundant dynamic point clouds, which contaminate static bed feature data and lead to clustering offset.
4. **Non-standard hardware installation**: Improper installation height or tilt angle of individual devices limits radar coverage of the sleeping area, resulting in inherent excessive localization errors.

These positioning errors mainly originate from complex environmental interference and non-standard hardware deployment, rather than algorithmic defects. Future optimization can effectively reduce such errors by standardizing radar installation specifications and introducing multipath filtering algorithms.

### 5.2. Limitations and Future Work

This section systematically analyzes the inherent limitations of the proposed framework, discusses their practical impacts on system performance, and clarifies targeted future research directions.

#### 5.2.1. Limitations of Evaluation Metrics

This study adopts return air temperature fluctuation as the core evaluation indicator, which is highly consistent with the research objective of stabilizing bedroom sleeping thermal environments. Nevertheless, per ASHRAE thermal comfort criteria, single temperature metrics cannot fully characterize comprehensive indoor thermal performance. Restricted by the hardware limitations of mass-produced commercial air conditioners, this work does not quantitatively evaluate airflow uniformity, local cold draft, energy consumption, and operating noise. However, temperature stability is the dominant factor affecting sleeping thermal comfort, and the unevaluated auxiliary indicators exert negligible impacts on the core control performance. Future work will establish a multi-dimensional evaluation framework to realize comprehensive performance verification.

#### 5.2.2. Incompleteness of Model Input Features

The LGBM model takes bed spatial coordinates, return air temperature, compressor frequency, and temperature deviation as core input features, which are highly correlated with bedroom thermal variation and airflow characteristics. To maintain the zero-extra-sensor deployment advantage, additional environmental parameters, including outdoor temperature, indoor humidity, room dimensions, and real-time occupant status, are not incorporated into the model. In typical residential bedroom scenarios, mild thermal load fluctuations only cause slight performance degradation, enabling the algorithm to maintain stable and reliable operating performance under daily working conditions. Subsequent research will fuse multi-source sensing data to enrich feature dimensions and improve model environmental adaptability and generalization ability.

#### 5.2.3. Performance Imbalance of Multi-Task Prediction

The multi-task model exhibits unbalanced prediction performance: louver control achieves accuracy and F1-scores above 0.98, while fan speed classification yields an accuracy of 0.74 and an F1-score of 0.72. This discrepancy stems from inherent task differences. Louver adjustment is a simple binary classification task with fixed control logic, whereas six-class fan speed prediction is susceptible to dynamic thermal loads, compressor operating fluctuations, and diversified user habits.

A comprehensive analysis of system operation and thermal comfort criteria verifies the rationality and engineering practicability of such performance imbalance:

1. **Steady-state temperature control robustness**: Louver-adjusted airflow direction dominates indoor temperature field reconstruction, while fan speed only undertakes fine-grained air volume tuning. Minor fan speed prediction errors cause negligible airflow changes and cannot undermine the system’s superior steady-state temperature stability.
2. **Thermal comfort sustainability**: In static sleeping scenarios, human thermal perception is primarily sensitive to ambient temperature and airflow direction. Slight airflow velocity deviations caused by fan speed prediction errors are imperceptible and will not degrade overall sleeping thermal comfort.
3. **Closed-loop system stability**: The temperature feedback closed-loop framework possesses inherent error correction capability. Abnormal fan speed outputs are timely adjusted via real-time thermal feedback, ensuring stable system operation without frequent regulation or operational surge.
4. **Engineering applicability**: Temperature stability and sleeping thermal comfort are the primary performance indicators for bedroom air conditioners. High-precision louver control guarantees core system performance, and fan speed prediction deviation is a secondary factor constrained by the closed-loop regulation mechanism.

In summary, the relatively low fan speed prediction accuracy is only a local performance deficiency and does not restrict the practical engineering application of the proposed method. Future work will adopt multi-model fusion and prior rule constraint strategies to optimize the fan speed prediction module and further improve overall system performance.

## 6. Conclusions

This paper proposes a spatial adaptive intelligent control algorithm integrating millimeter-wave radar bed localization and an LGBM weak teacher model to address excessive steady-state temperature fluctuations of residential air conditioners after setpoint attainment. Different from traditional passive temperature-error-based control strategies, the proposed method takes bed spatial position as the core feature to characterize indoor sleeping comfort zones, and optimizes louver angle and fan speed through a large-scale cloud-trained LGBM model, thereby effectively suppressing indoor temperature oscillation.

Extensive experiments validate the effectiveness and practicality of the proposed method. Large-scale field tests on 719 devices achieve an 83.6% bed localization consistency rate within a 0.5 m tolerance range, and laboratory tests realize a centimeter-level average localization error of 0.255 m. The multi-task control model exhibits stable convergence and outstanding steady-state performance, reducing $MAF_{T_{1}}$ and $VAR_{T_{1}}$ to 0.21 °C and $0.064\;{}^{\circ}C^{2}$, respectively. The model maintains ultra-high louver control precision and acceptable fan speed prediction accuracy, fully meeting practical deployment requirements.

This work realizes the effective fusion of spatial perception and data-driven air conditioning control, providing a low-cost, high-universality, unattended optimization solution for improving residential thermal stability. It validates the critical value of sleeping area spatial information in indoor thermal regulation, offering a novel technical reference for human-centered intelligent thermal comfort control systems. Future research will focus on multi-source feature fusion, multi-dimensional performance evaluation, and multi-task model optimization to further enhance the adaptive capability and comprehensive performance of the proposed control system.

## Figures and Tables

**Table 1:** Core Hyperparameters of the Model.

Sub-Module	Number of Decision Trees	Maximum Tree Depth	Learning Rate	Regularization Coefficient λ	Regularization Coefficient γ
Large louver classification sub-model	100	6	0.05	0.01	0.1
Small louver classification sub-model	100	6	0.05	0.01	0.1
Fan speed classification sub-model	90	6	0.05	0.01	0.1

**Table 2:** Comparison of localization errors among different clustering algorithms.

	Algorithm Result	Ground Truth	Eabs (Proposed)	Eabs (DBSCAN)	Eabs (K-Means)	Eabs (Mean-Shift)
A	(2.9 m, 125.1°)	(3 m, 120°)	0.281	0.323	0.485	0.563
B	(2.5 m, 101.5°)	(2.7 m, 95°)	0.356	0.513	0.486	0.505
C	(2.17 m, 62°)	(2.3 m, 68°)	0.238	0.548	0.613	0.379
D	(1.1 m, 74.4°)	(1.1 m, 84°)	0.144	0.245	0.511	0.158
Average	–	–	0.255	0.407	0.449	0.439

**Table 3:** Ablation Experimental Results.

Experiment	${\mathrm{MAF}}_{T_{1}}$ (°C)	${\mathrm{NFR}}_{T_{1}}$ (%)	Avg Accuracy
full	0.2177	21.47	0.9342
no_pos	0.5027	7.64	0.8549
no_reg	0.4981	8.55	0.9049
no_$T_{1}$	0.7841	2.12	0.7926
no_f	0.3054	16.23	0.9015
no_$\Delta T_{\mathrm{set}}$	0.2763	18.05	0.9137

**Table 4:** Performance Comparison of Various AC Control Strategies in Real Tests.

Control Strategy	${\mathrm{MAF}}_{T_{1}}$ (°C)	${\mathrm{VAR}}_{T_{1}}$ (°C^2^)	${\mathrm{NFR}}_{T_{1}}$ (%)
Original Factory Automatic Control	0.5732	0.3143	2.6846
Traditional PID Control	0.6615	0.3217	3.1273
XGBoost Single-Task Control	0.4813	0.2802	6.2457
Vanilla Single-Task LGBM	0.4286	0.2094	7.8912
Proposed Algorithm Control	0.1884	0.0501	13.5922

## Data Availability

The original contributions presented in this study are included in the article. Further inquiries can be directed to the corresponding authors.

## Abbreviations

The following abbreviations are used in this manuscript:

LGBM	Light Gradient Boosting Machine
MEC	Minimum Enclosing Circle
CDF	Cumulative Distribution Function
ASHRAE	American Society of Heating, Refrigerating and Air-Conditioning Engineers
PID	Proportional-Integral-Derivative
GOSS	Gradient-based One-side Sampling
GBDT	Gradient Boosting Decision Tree
MAF	Mean Absolute Fluctuation
NFR	No Fluctuation Ratio
